# Fibroblast growth factor 2 is an essential cardioprotective factor in a closed‐chest model of cardiac ischemia‐reperfusion injury

**DOI:** 10.14814/phy2.12278

**Published:** 2015-01-27

**Authors:** Stacey L. House, Joy Wang, Angela M. Castro, Carla Weinheimer, Attila Kovacs, David M. Ornitz

**Affiliations:** Division of Emergency Medicine, Washington University in St. Louis School of Medicine, St. Louis, Missouri, USA; Department of Developmental Biology, Washington University in St. Louis School of Medicine, St. Louis, Missouri, USA; Center for Cardiovascular Research, Department of Medicine, Washington University in St. Louis School of Medicine, St. Louis, Missouri, USA

**Keywords:** Cardiac repair, cardioprotection, FGF2, fibroblast growth factor, ischemia‐reperfusion injury, myocardial infarction

## Abstract

Fibroblast growth factor 2 (FGF2) is cardioprotective in in vivo models of myocardial infarction; however, whether FGF2 has a protective role in in vivo ischemia‐reperfusion (IR) injury, a model that more closely mimics acute myocardial infarction in humans, is not known. To assess the cardioprotective efficacy of endogenous FGF2, mice lacking a functional *Fgf2* gene (*Fgf2*^−/−^) and wild‐type controls were subjected to closed‐chest regional cardiac IR injury (90 min ischemia, 7 days reperfusion). *Fgf2*^−/−^ mice had significantly increased myocardial infarct size and significantly worsened cardiac function compared to wild‐type controls at both 1 and 7 days post‐IR injury. Pathophysiological analysis showed that at 1 day after IR injury *Fgf2*^−/−^ mice have worsened cardiac strain patterns and increased myocardial cell death. Furthermore, at 7 days post‐IR injury, *Fgf2*^−/−^ mice showed a significantly reduced cardiac hypertrophic response, decreased cardiac vessel density, and increased vessel diameter in the peri‐infarct area compared to wild‐type controls. These data reveal both acute cardioprotective and a longer term proangiogenic potential of endogenous FGF2 in a clinically relevant, in vivo, closed‐chest regional cardiac IR injury model that mimics acute myocardial infarction.

## Abstract (precis)

The cardioprotective efficacy of endogenous FGF2 was tested using a closed‐chest regional cardiac IR injury model. Mice lacking FGF2 (*Fgf2*^−/−^) mice had significantly increased myocardial infarct size and significantly worsened cardiac function compared to wild‐type controls at both 1 and 7 days post‐IR injury. These data reveal both acute cardioprotective and a longer term proangiogenic potential of endogenous FGF2 in a clinically relevant, in vivo, closed‐chest regional cardiac IR injury model that mimics acute myocardial infarction.

## Introduction

Ischemic heart disease is the leading cause of death worldwide resulting in 7.4 million deaths in 2012 (World Health Organization 2014). In the United States alone, approximately one million acute myocardial infarctions occur each year (American Heart Association 2010). Current therapy for acute myocardial infarction relies primarily on early reperfusion strategies such as the use of lytics, percutaneous coronary interventions, and coronary artery bypass graft surgery for patients with multivessel disease. While reperfusion therapy is necessary to limit the cell death resulting from cardiac ischemia, reperfusion in and of itself results in damage to the myocardium through the activation of reactive oxygen species, inflammation, and endothelial damage. Together, cardiac ischemia‐reperfusion (IR) injury causes cardiac dysfunction (i.e., myocardial stunning), cardiomyocyte death (i.e., infarction), and remodeling of cardiac tissues (i.e., cardiomyocyte hypertrophy, fibrosis, and vascular remodeling).

Many studies have shown that stimuli such as brief ischemic insults, pharmacologic agents, and endogenous proteins including Fibroblast Growth Factors (FGFs) can elicit protection from ischemia‐reperfusion injury (Murry et al. 1986; Murphy and Steenbergen 2008). The FGF family consists of 18 signaling proteins that regulate cell growth and differentiation, angiogenesis, tissue homeostasis, and tissue repair (Ornitz and Itoh 2001; Schultz 2002). A member of this family, FGF2, or basic FGF, is one of the most predominantly expressed FGFs in the heart and is expressed in all developmental stages and in several cell types including cardiomyocytes, endothelial cells, smooth muscle cells, and fibroblasts (Kardami et al. 2007). In addition, FGF2 is upregulated in the heart during various forms of cardiac pathology including ischemic damage and cardiac hypertrophy (Detillieux et al. 2004). Pharmacologic and in vitro studies suggest that FGF2 is cardioprotective (Detillieux et al. 2004). Additionally, cardiac‐specific overexpression of human FGF2 results in decreased postischemic cardiac dysfunction and myocardial infarction, whereas ablation of the *Fgf2* gene results in worsened postischemic outcomes in an *ex vivo* work‐performing murine model of global IR injury (House et al. 2003). Ablation of the *Fgf2* gene also decreases fibroblast proliferation, decreases collagen deposition, decreases scar contraction, and results in increased infarct size after chronic cardiac ischemia (Virag et al. 2007). In addition, human recombinant FGF2 given during early reperfusion injury in *ex vivo* models is cardioprotective (Jiang et al. 2009; House et al. 2010a).

Previous studies highlight the potential for therapeutic use of FGF2 in cardioprotection. However, these studies have primarily focused on *ex vivo* isolated heart IR or chronic permanent coronary occlusion. In vivo characterization of the cardioprotective efficacy of FGF2 in IR injury is necessary to understand its cardioprotective potential in a clinically relevant model. In addition, future therapeutic use of FGF2 for acute myocardial infarction would likely focus on activation of FGF2 signaling during reperfusion of the myocardium. One of the primary mediators of reperfusion injury is activation of inflammatory cascades. Current in vivo techniques of open chest cardiac IR injury involve massive activation of inflammatory cascades as a result of acute surgical manipulation that can mask the important contributions of inflammation to the reperfusion injury that occurs in patients. To circumvent these problems, we have utilized a newer methodology of closed‐chest IR injury, which removes the artificial activation of inflammation from surgical thoracotomy that would not otherwise occur in humans suffering an acute myocardial infarction.

To determine the function(s) of endogenous FGF2 in cardiac IR injury, we used echocardiography to assess the acute requirement of FGF2 for cardiac functional recovery and infarct size after IR injury to assess the role of FGF2 during subsequent cardiac remodeling. Histological methods were used to evaluate the function of FGF2 in the cardiac hypertrophic response and vascular remodeling after IR injury. We find that endogenous FGF2 mediates cell survival, postischemic functional recovery, vascular remodeling, and the cardiac hypertrophic response in a closed‐chest model of cardiac ischemia‐reperfusion injury.

## Methods

### Mice

Mice were housed in a specific pathogen‐free facility and handled in accordance with standard use protocols, animal welfare regulations, and the *NIH Guide for the Care and Use of Laboratory Animals*. All protocols were approved by the Washington University Animal Studies Committee. Mice with a targeted ablation of the *Fgf2* gene (*Fgf2*^−/−^) maintained on a C57BL/6J, 129X1 mixed background were generated as previously described (Zhou et al. 1998). Wild‐type and *Fgf2*^−/−^ mice (both male and female) at 8 to 10 weeks of age were randomly assigned to the present study – 90 min ischemia + 1 day reperfusion, sham IR surgery + 1 day recovery, 90 min ischemia + 3 days reperfusion, sham IR surgery + 3 days recovery, 90 min ischemia + 7 days reperfusion, or sham IR surgery + 7 days recovery. There were no significant differences observed between male and female mice with respect to the effect of *Fgf2* ablation on any of the studied outcomes. All data depicted include a mix of both male and female mice.

### Mouse model of closed‐chest cardiac ischemia‐reperfusion injury

**Figure 1.**
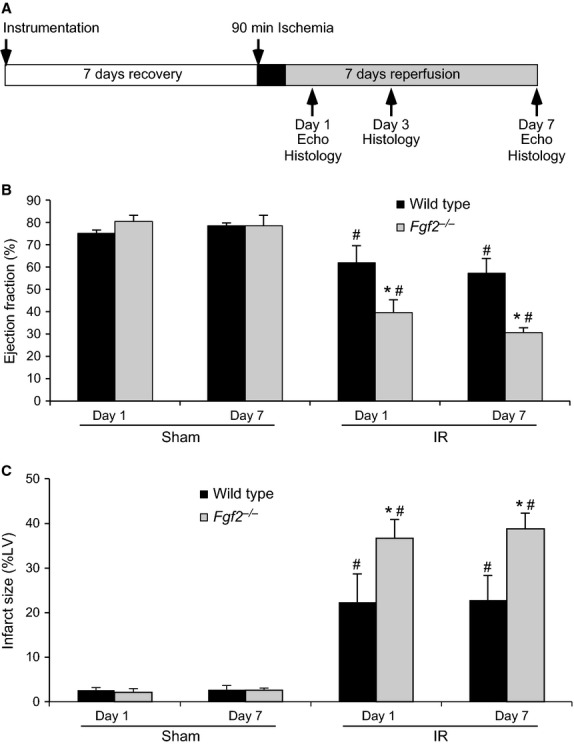
Endogenous FGF2 improves cardiac functional recovery and reduces wall motion abnormalities after closed‐chest cardiac ischemia‐reperfusion injury. (A) Schematic of closed‐chest ischemia‐reperfusion protocol. (B) *Fgf2*^−/−^ mice show significantly decreased cardiac function (ejection fraction) compared to wild‐type controls at both 1 day and 7 days after IR injury. (C) *Fgf2*^−/−^ mice have increased infarct size (determined by wall motion abnormalities viewed by echocardiography) at 1 day and 7 days after IR injury. *n* = 4–7, *P < 0.05 versus wild type, ^#^*P* < 0.05 versus sham.

The mouse model of closed‐chest cardiac IR injury was adapted from previous studies (Nossuli et al. 2000) and performed in the Mouse Cardiovascular Phenotyping Core at Washington University in St. Louis School of Medicine (Fig. 1A). Wild‐type and *Fgf2*^−/−^ mice at 8–10 weeks of age were anesthetized with ketamine/xylazine (100 mg/kg and 10 mg/kg, i.p.). Mice were surgically prepped and ventilated through a tracheostomy. Mice were then subjected to a left mini‐thoracotomy and the pericardium was dissected. Then, an 8‐0 polypropylene suture with a U‐shaped tapered needle was passed under the proximal left anterior descending (LAD) artery. The needle was cut from the suture and the two ends of the 8‐0 suture were threaded through a 0.5 mm piece of PE‐10 tubing that had been previously soaked for 24 h in 100% ethanol. The suture formed a loose snare around the LAD. Each end of the suture was then threaded through the end of a size 3 Kalt needle and exteriorized through each side of the chest wall and the chest wall was closed. The ends of the exteriorized 8‐0 suture were tucked under the skin and the skin closed. The mouse was removed from the respirator and allowed to recover for 7 days. After this recovery time, mice were re‐anesthetized but not ventilated, and only the skin above the chest wall was reopened. Mice were taped to an EKG board (Indus Instruments) to observe ST segment changes during ischemia and reperfusion. The suture ends were pulled apart gently until ST segment elevation appeared on the EKG indicating LAD occlusion. The sutures were then secured in this position and ischemia was continued for 90 min. To induce reperfusion, the sutures were cut close to the chest wall releasing the tension, and reperfusion was confirmed with resolution of ST segment elevation. Mice assigned to sham surgery, received the same instrumentation surgery as mice assigned to IR injury and then were allowed to recover for 7 days. For sham‐treated mice, the surgical manipulation on the day of IR injury was performed the same as for mice assigned to receive IR injury, except the ends of the suture encircling the LAD were not pulled apart. Sham‐treated mice had the same length of anesthesia, ventilation, and other surgical manipulation as mice subjected to IR injury. A mixture of genotypes and sham or IR treatments was performed on each surgical day to reduce the risk of variability. The surgeon was blinded to mouse genotype for all interventions, and assessors of all cardiac endpoints were blinded to genotype and treatment group. The overall mortality for the closed‐chest ischemia‐reperfusion model was 8.4% (10/119) with all deaths occurring after the instrumentation surgery before the onset of ischemia. There was no significant effect of genotype on mortality rate (8.8% for wild‐type, 8.1% for *Fgf2*^−/−^ mice).

### Echocardiography

Mouse echocardiography was performed in the Washington University Mouse Cardiovascular Phenotyping Core facility using a Visual Sonics Vevo 2100 High‐Resolution In Vivo Imaging System. To obtain images, mice were anesthetized with Avertin (2,2,2‐tribromoethanol, 250 mg/kg, i.p.), which was chosen due to its lack of cardiovascular effects at the doses used. High‐resolution echocardiography (VisualSonics) was utilized for phenotypic analysis of cardiac function and infarct area. Ejection fraction was calculated by measuring the end systolic and diastolic volumes from long‐axis images using the following formula: 100 * (LV end diastolic volume − LV end systolic volume/LV end diastolic volume). Quantitative speckle tracking echocardiography was performed using the VevoStrain software (Visual Sonics) as previously described (Vyas et al. 2012). Infarct area was calculated by examining serial short‐axis images (1 mm apart) spanning from the base of the left ventricle (level of the aortic valve) to the apex. The percent area of the ventricle that was infarcted was calculated by determining the percent of the ventricle that was hypokinetic (Kanno et al. 2002; Lavine et al. 2013).

### Histological examination

Wild‐type and *Fgf2*^−/−^ mice subjected to cardiac IR injury were anesthetized and the heart was removed from the thoracic cavity. Hearts were rinsed in phosphate‐buffered saline and halted in diastole in potassium chloride‐saturated phosphate‐buffered saline. Hearts were then fixed in 10% formalin and transferred to 70% ethanol the next day. Hearts were paraffin‐embedded and then sectioned transversely (10 *μ*m). Hematoxylin/eosin and Masson's trichrome stain were used for histological and pathological examination of hearts.

#### Measurement of myoctye cross‐sectional area

Sections were deparaffinized, rehydrated, and stained with fluorescent FITC‐labeled wheat germ agglutinin (1:4 dilution, Sigma‐Aldrich, St. Louis, MO) and counterstained with DAPI to show nuclei (Vector Laboratories, Burlingame, CA). Fluorescence was visualized using a Zeiss ApoTome microscope, and multiple digital images from the peri‐infarct and remote (LV septum) area were captured and processed with AxioVision Release 4.8 software. Using NIH Image J software, at least 150 cells per heart were measured to determine the average myocyte cross‐sectional area for each heart.

#### Measurement of blood vessel density and size

Heart sections were deparaffinized, rehydrated, and stained with fluorescent TRITC‐labeled antismooth muscle actin antibody (1:100 dilution, Sigma‐Aldrich) and counterstained with DAPI to show nuclei (Vector Laboratories). Fluorescence was visualized using a Zeiss ApoTome microscope as described above. The number of smooth muscle actin‐positive vessels per 10X microscopic field and the average vessel diameter were determined in at least 16 fields adjacent to the infarct from each heart (6 different sections) using AxioVision Release 4.8 software.

#### Measurement of capillary density and size

Heart sections were deparafinized, rehydrated, and subjected to antigen retrieval in pH 6.0 citrate buffer in a pressure cooker for 10 min. Sections were then permeabilized in 0.1% Triton/PBS for fifteen minutes. Sections were blocked with 10% goat serum for 30 min followed by incubation with rat anti‐CD31 antibody (1:20 dilution, Dianova, Hamburg, Germany) overnight at 4°C. Cy3 conjugated anti‐rat IgG (1:500 dilution, Sigma‐Aldrich) was then applied for 1 h at room temperature. To decrease autofluorescence of the cardiac tissue, sections were treated with 0.1% Sudan Black (Sigma‐Aldrich) dissolved in 70% ethanol for 30 min. Sections were counterstained with DAPI (Vector Laboratories) and imaged with a Zeiss ApoTome microscope. At least 16 images (20X) in the peri‐infarct region were obtained from each heart (from six sections). NIH Image J software was utilized to determine the number of capillaries, average capillary size, and number of nuclei per field.

#### Measurement of TUNEL‐positive nuclei

Heart sections were subjected to the DeadEND Fluorometric TUNEL Assay (Promega, Madison, WI) according to the package insert. Sections were counterstained with DAPI (Vector Laboratories) and imaged with a Zeiss Apotome microscope. At least 16 images (20X) in the peri‐infarct region were obtained from each heart (from six sections). NIH Image J software was utilized to determine the number of TUNEL‐positive nuclei and number of nuclei per field.

### Quantitative RT‐PCR

Wild‐type and *Fgf2*^−/−^ mice subjected to cardiac IR injury or sham ischemia were anesthetized and the heart was removed. The left ventricle was quickly dissected into the IR area and the remote area (LV septum). These portions of the heart were snap frozen in liquid nitrogen and stored at −80°C. RNA was extracted using a Qiagen RNeasy Minikit (Qiagen, Valencia, CA, USA), with on‐column DNA digest. RNA concentration was determined with a Nanodrop spectrophotometer. cDNA was made using the BioRad iScript Reverse Transcription Supermix for qRT‐PCR (BioRad, Hercules, CA, USA). qRT‐PCR was performed on an Applied Biosystems (ABI 7500 machine) using ABI Taqman Fast Advanced Master Mix and Taqman gene expression assays for *Hprt*,* Gapdh*,* Fgf2*,* Fgf9*, or *Fgf16*. All samples were normalized to *Hprt* and then scaled relative to the sham‐treated cohort using the standard ddCT method. An additional set of samples at 7 days after IR injury subjected to qRT‐PCR was normalized to *Gapdh* using the ddCT method.

### Statistical analysis

All values are expressed as mean ± standard error of the mean. Echocardiography data for LV infarct size and ejection fraction were compared using analysis of variance with a post hoc Student's *t‐*test. The remaining data were compared using a Student's *t*‐test. Data with a *P* < 0.05 were considered statistically significant.

## Results

To determine the cardioprotective efficacy of endogenous FGF2, we have utilized mice with a targeted ablation of the *Fgf2* gene (*Fgf2*^−/−^ mice) (Zhou et al. 1998). *Fgf2*^−/−^ mice are viable and fertile. They exhibit no baseline alteration in cardiac function or morphometry as assessed by echocardiography (House et al. 2010b) and hemodynamic measurements (House et al. 2003). *Fgf2*^−/−^ and wild‐type mice at 8–10 weeks of age were subjected to a closed‐chest cardiac IR injury model. Cardiac function was analyzed at 1 day and 7 days after IR injury using echo analysis of long‐axis views of the left ventricle (Fig. 1A). These time points were chosen to characterize acute effects after IR injury as well as changes occurring during ventricular remodeling. *Fgf2*^−/−^ mice showed a significant reduction in ejection fraction at both 1 day and 7 days after IR injury compared to wild‐type controls (Fig. 1B and Video S1, *P* < 0.05) suggesting the requirement of endogenous FGF2 in the recovery of contractile function after IR injury.

**Figure 2.**
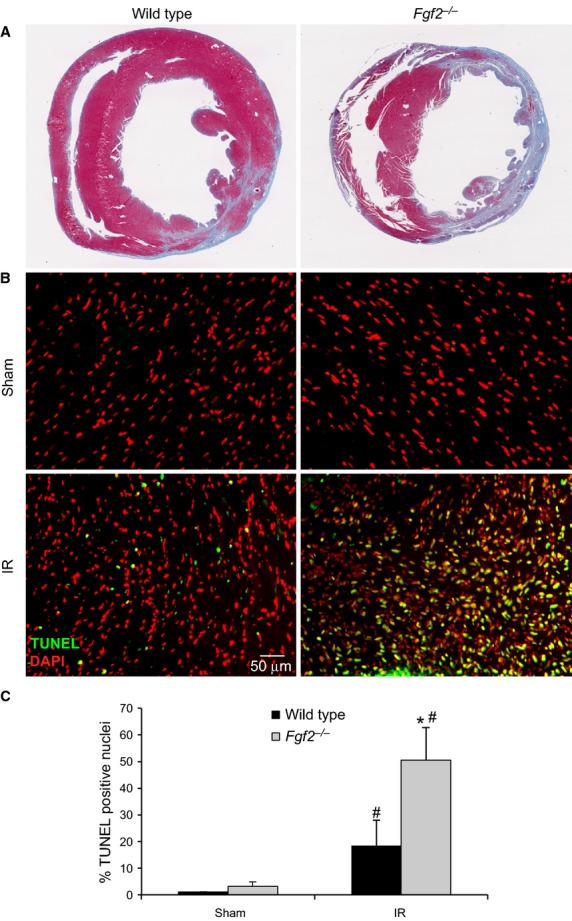
Endogenous FGF2 reduces cell death and infarct size resulting from cardiac ischemia‐reperfusion injury. (A) Representative images of Masson's Trichrome‐stained *Fgf2*^−/−^ and wild‐type mice 7 days after IR injury. (B) Representative images of TUNEL assays of *Fgf2*^−/−^ and wild‐type mice 1 day after IR injury or sham treatment (green – TUNEL positive nuclei, red – DAPI). (C) *Fgf2*^−/−^ mice have increased cell death (% TUNEL positive nuclei) at 1 day after IR injury compared to wild‐type controls. *n* = 3–5, *P < 0.05 versus wild type, ^#^*P* < 0.05 versus sham.

Echocardiography was also used to determine the size of the infarct area in *Fgf2*^−/−^ and wild‐type mice subjected to closed‐chest IR injury by measuring the hypokinetic area in serial short‐axis views though the left ventricle. This methodology has been validated and compared to histologic analysis of myocardial infarct size (Kanno et al. 2002). *Fgf2*^−/−^ mice showed significantly increased infarct size as a percent of the left ventricle compared to wild‐type controls after IR injury, both acutely (1 day post‐reperfusion) and during the remodeling process (7 days post‐reperfusion) (Fig. 1C and Video S2, *P* < 0.05). Trichrome staining, a marker of fibrosis, also showed increased scar area 7 days after IR injury in the *Fgf2*^−/−^ mice compared to wild‐type mice (wild‐type 27 ± 5%, *Fgf2*^−/−^ 45 ± 6%, *P* < 0.05, representative images Fig. 2A). Consistent with our finding of acutely increased infarct size by echocardiography 1 day after IR injury, TUNEL assay showed increased cell death in the infarct area of *Fgf2*^−/−^ hearts compared to wild‐type controls (Fig. 2B and C, *P* < 0.05).

**Figure 3.**
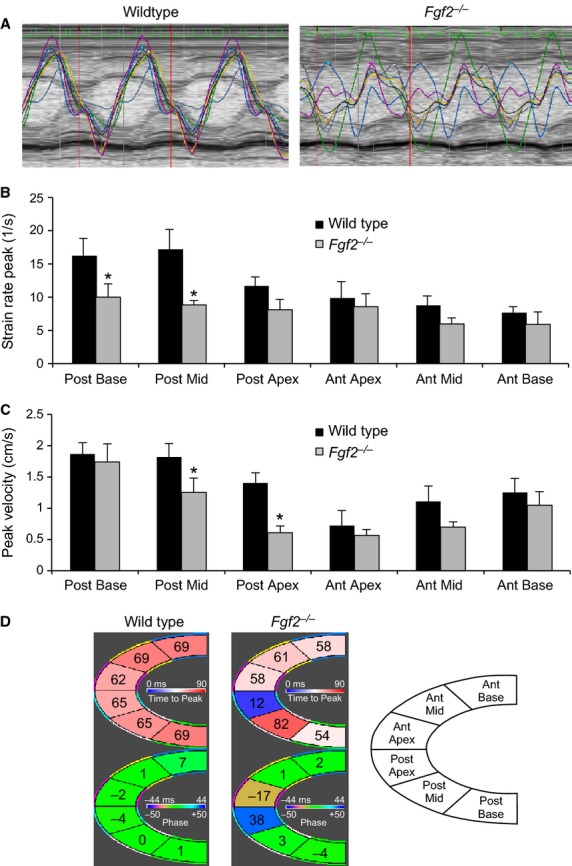
Endogenous FGF2 reduces left ventricular segmental cardiac dysfunction and cardiac dyssynchronicity. (A) Representative regional radial strain rate curves representing six segments of the left ventricle in wild‐type and *Fgf2*^−/−^ mice 7 days after IR injury. *Fgf2*^−/−^ mice have regionally decreased radial peak strain rate (B) and radial peak velocity (C) compared to wild‐type controls, 7 days after IR injury. (D) Representative segmental time to peak strain rate (top) and phase delay (bottom) in wild‐type and *Fgf2*^−/−^ mice 7 days after IR injury. *Fgf2*^−/−^ mice have more variable time to peak strain rate values and increased phase delay near the apex compared to wild‐type controls suggesting increased postischemic dyssynchronicity. Anterior wall corresponds to LV septum (remote area); posterior wall corresponds to LV free wall (IR area). *n* = 6, *P < 0.05 versus wild type.

Speckle tracking echocardiography was also used to further characterize segmental wall motion abnormalities observed in *Fgf2*^−/−^ hearts after IR injury. Both longitudinal and radial strain measures were performed, as longitudinal strain is most reflective of myocardial shortening at the level of the endocardium and radial strain is most reflective of myocardial shortening at the level of the mid‐myocardium (Bauer et al. 2011). Consistent with our observations of decreased ejection fraction in *Fgf2*^−/−^ hearts after IR injury, we observed significantly decreased global radial and longitudinal peak strain rate in *Fgf2*^−/−^ hearts at both 1 day and 7 days after IR injury (Table 1). Global peak displacement and peak strain showed significant worsening between 1 day and 7 days after IR injury in both wild‐type and *Fgf2*^−/−^ hearts, but there was no significant difference between wild‐type and *Fgf2*^−/−^ hearts. Due to the localized damage caused by IR injury, speckle tracking echocardiography was used to quantify the relative function of different portions of the left ventricle. Representative tracings of peak radial strain rate of six individual segments of the left ventricle 7 days after IR injury are shown in Figure 3A. *Fgf2*^−/−^ hearts showed less coordinated myocardial strain among the left ventricular segments than wild‐type hearts 7 days after IR injury. To determine if postischemic cardiac dysfunction in *Fgf2*^−/−^ hearts was primarily related to the markedly decreased function in the most affected region of the left ventricle (e.g., the apex) or more a result of widespread myocardial dysfunction in *Fgf2*^−/−^ mice, we determined peak radial velocity and peak radial strain rate at various regions of the left ventricle. This analysis showed similar dysfunction (strain rate and velocity) of the apex in wild‐type and *Fgf2*^−/−^ mice after IR injury (Fig. 3B and C). In contrast, *Fgf2*^−/−^ mice had a significantly decreased peak radial strain rate in the posterior wall extending from the apex to the base (Fig. 3B). *Fgf2*^−/−^ mice also had significantly decreased peak velocity in the mid ventricular segments of the anterior and posterior walls and posterior apex compared to wild‐type controls (Fig. 3C). Left ventricular synchronicity is an important contributor to overall cardiac function and can be reduced in IR injury. An established method of calculating wall delay was used to determine left ventricular synchronicity at 7 days after IR injury (Eguchi et al. 2012). This method divides the left ventricle into six separate segments and calculates the difference in the time it takes for each segment of the left ventricle to reach its peak strain (Fig. 3D). *Fgf2*^−/−^ mice showed increased maximum opposing wall delay 7 days after IR injury compared to wild‐type controls, suggesting worsened global dyssynchronicity (wild type 32.4 ± 8.1 ms vs. *Fgf2*^−/−^ 57.8 ± 9.8 ms, *P* < 0.05). Representative segmental time to peak strain and segmental phase delay (difference in time between peak strain rate of each sector and average left ventricular peak strain rate) showed increased dyssynchronicity in *Fgf2*^−/−^ hearts 7 days after IR injury primarily in the apical and mid posterior wall (Fig. 3D).

**Table 1.** Global left ventricular myocardial strain analysis.

		Radial		Longitudinal	
		Wild type	FGF2^−/−^	Wild type	FGF2^−/−^
Velocity peak (cm/s)	IR Day 1	1.0 ± 0.3	1.0 ± 0.1	1.3 ± 0.3	0.7 ± 0.04
	IR Day 7	1.4 ± 0.2	1.0 ± 0.2	1.1 ± 0.1	0.8 ± 0.1
Displacement peak (mm)	IR Day 1	0.3 ± 0.03	0.2 ± 0.1	0.2 ± 0.04	0.1 ± 0.03
	IR Day 7	0.1 ± 0.1^#^	0.1 ± 0.40^#^	0.1 ± 0.04^#^	0.1 ± 0.04
Strain peak (%)	IR Day 1	24.0 ± 3.4	24.4 ± 3.4	18.6 ± 6.8	12.2 ± 1.2
	IR Day 7	11.2 ± 6.9^#^	8.6 ± 2.2^#^	5.9 ± 3.1^#^	8.1 ± 2.3^#^
Strain rate peak (%)	IR Day 1	12.3 ± 0.6	7.2 ± 0.4*	11.2 ± 3.9	5.7 ± 0.3*
	IR Day 7	11.9 ± 1.3	7.9 ± 1.2*	9.9 ± 1.8	6.7 ± 0.8*

Data are mean ± SEM. *n* = 3–6, *P < 0.05 versus wild type, ^#^*P* < 0.05 versus 1 day time point.

FGF2 has been shown to mediate cardiac hypertrophy in response to multiple stimuli including pressure overload (Schultz et al. 1999), angiotensin II (Pellieux et al. 2001), and isoproterenol (House et al. 2010b). For comparison with other models, the cardiac hypertrophic response was evaluated during remodeling after IR injury. Heart weight versus body weight ratios showed no significant differences between *Fgf2*^−/−^ or wild‐type hearts 7 days after IR injury (in mg heart weight/g body weight, 7.6 ± 0.8 for wild type, 7.1 ± 0.2 for *Fgf2*^−/−^).

**Figure 4.**
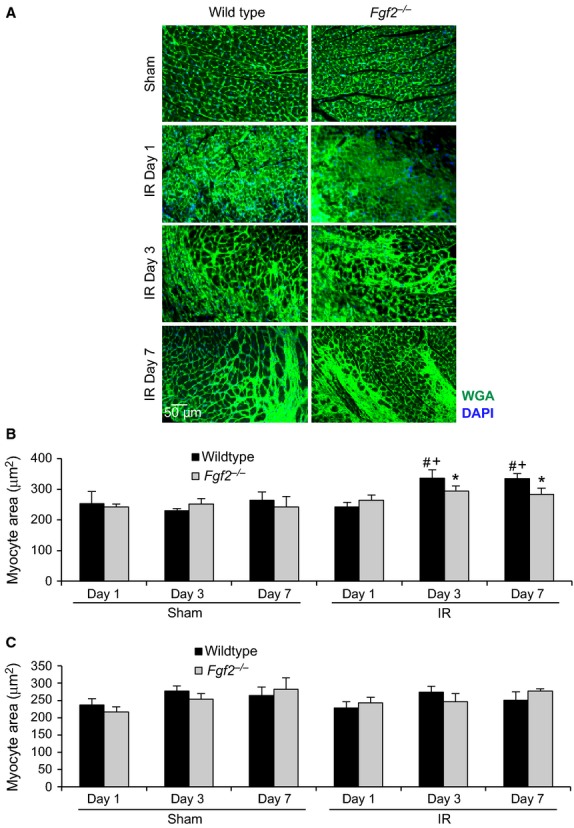
Endogenous FGF2 mediates the cardiac hypertrophic response in the peri‐infarct area after cardiac ischemia‐reperfusion injury. (A) Representative images of wheat germ agglutinin staining in the peri‐infarct area of *Fgf2*^−/−^ and wild‐type mice at multiple time points after IR injury or sham treatment (green – wheat germ agglutinin, blue – DAPI). (B) *Fgf2*^−/−^ mice have decreased cardiomyocyte cross‐sectional area in the peri‐infarct area at 3 and 7 days after IR injury compared to wild‐type controls. (C) There is no significant difference in cardiomyocyte cross‐sectional area in the remote myocardium in any treatment group. *n* = 3–8, *P < 0.05 versus wild type, ^#^*P* < 0.05 versus sham, ^+^*P* < 0.05 versus 1 day time point.

Since the hypertrophic response in regional IR injury may be localized in the peri‐infarct area and not fully reflected in heart weight versus body weight ratio changes, the cardiac hypertrophic response was also measured at the cellular level. Fluorescently labeled wheat germ agglutinin was utilized to visualize cardiomyocyte size (Fig. 4A). There was no significant difference in cardiomyocyte cross‐sectional area in *Fgf2*^−/−^ hearts compared to wild‐type controls during cardiac remodeling after IR injury in the left ventricular septum, remote from the infarct (Fig. 4C). However, in the peri‐infarct area, *Fgf2*^−/−^ hearts had reduced cardiomyocyte size compared to wild‐type hearts at 3 days and 7 days after IR injury (Fig. 4B), demonstrating an impaired hypertrophic response in *Fgf2*^−/−^ hearts. *Fgf2*^−/−^ mice at 7 days after IR injury showed no difference in pulmonary edema compared to wild‐type mice as assessed by wet lung weight/body weight ratios (in mg wet lung weight/g body weight, 7.8 ± 0.4 for wild type, 7.6 ± 1.0 for *Fgf2*^−/−^) or wet lung weight/dry lung weight ratios (in mg wet lung weight/mg dry lung weight, 4.2 ± 0.1 for wild type, 4.1 ± 0.4 for *Fgf2*^−/−^).

**Figure 5.**
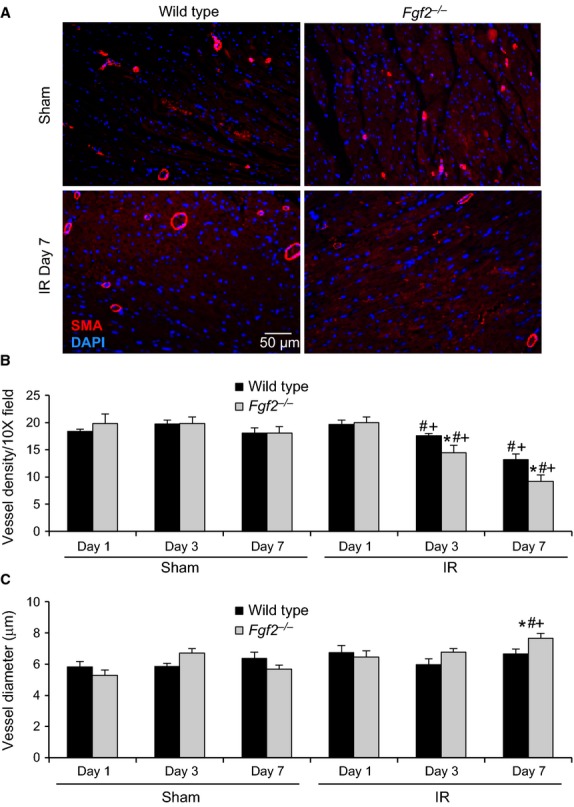
Endogenous FGF2 mediates vascular remodeling after cardiac ischemia‐reperfusion injury. (A) Representative images of smooth muscle actin‐positive vessels in *Fgf2*^−/−^ and wild‐type mice at 7 days after IR injury or sham treatment (red – smooth muscle actin, blue – DAPI). (B) *Fgf2*^−/−^ mice have decreased smooth muscle actin‐positive vessels in the peri‐infarct area at 3 and 7 days after IR injury compared to wild‐type controls. (C) *Fgf2*^−/−^ mice have increased average vessel diameter in the peri‐infarct area at 7 days after IR injury compared to wild‐type controls. *n* = 3–8, *P < 0.05 versus wild type, ^#^*P* < 0.05 versus sham, ^+^*P* < 0.05 versus 1 day time point.

**Figure 6.**
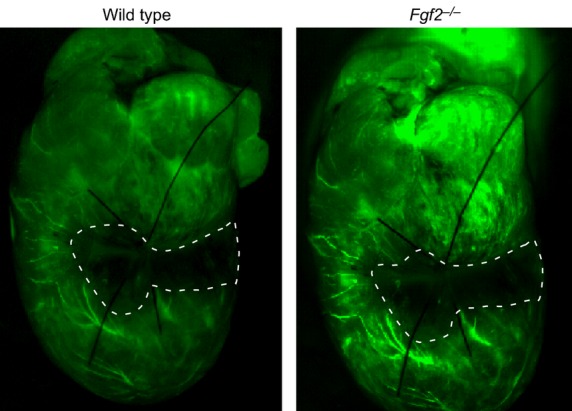
Representative whole mount images of FITC‐dextran injected *Fgf2*^−/−^ and wild‐type mice showing similar area at risk with left anterior descending artery ligation. The hypoperfused area is outlined in white dashed lines.

**Figure 7.**
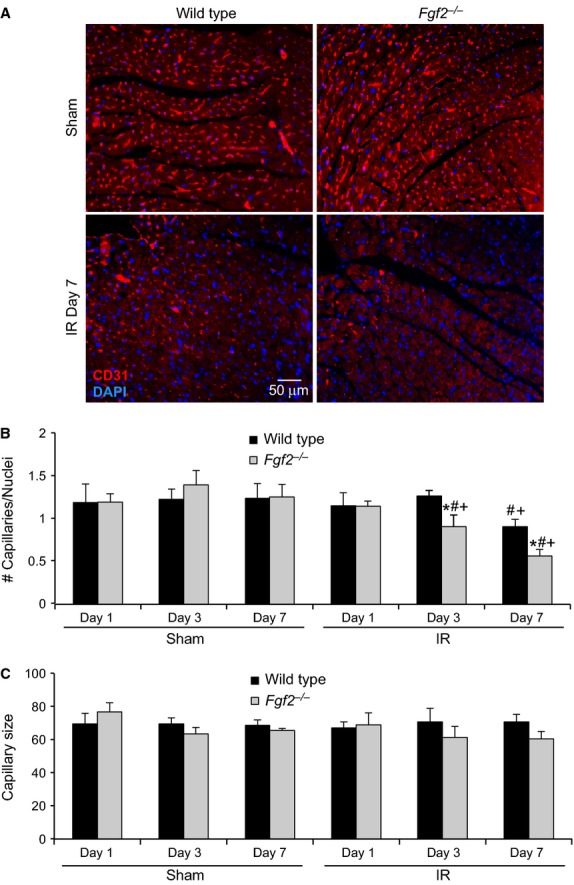
Endogenous FGF2 mediates capillary remodeling after cardiac ischemia‐reperfusion injury. (A) Representative images of capillaries in *Fgf2*^−/−^ and wild‐type mice at 7 days after IR injury or sham treatment (red – CD31, blue – DAPI). (B) *Fgf2*^−/−^ mice have decreased numbers of capillaries (normalized to number of cells present) in the peri‐infarct area at 3 and 7 days after IR injury compared to wild‐type controls. (C) There is no significant difference in capillary size in the peri‐infarct area in any treatment group. *n* = 3–8, *P < 0.05 versus wild type, ^#^*P* < 0.05 versus sham, ^+^*P* < 0.05 versus 1 day time point.

After IR injury in the heart, vascular remodeling occurs in which vessels in the ischemic zone are lost and are replaced by a process of neoangiogenesis (Tyagi 1997). Given the established role of FGF2 in the angiogenic response in multiple models of tissue injury (Goncalves 1998; Oladipupo et al. 2014), vessel density and vessel size were measured after cardiac IR injury. Noninfarcted *Fgf2*^−/−^ hearts showed no differences from wild‐type hearts with respect to vessel density and size (Fig. 5) (House et al. 2003). To determine if there was an effect of FGF2 ablation on the perfusion territory of the left anterior descending artery, which might affect our study, mice were injected intravenously with fluorescently labeled dextran after occlusion of the left anterior descending artery. Examination of the region of the heart lacking perfusion (Fig. 6) showed similar nonperfused area of the left ventricle between *Fgf2*^−/−^ and wild‐type hearts (29 ± 3% for wild type, 28 ± 3% for *Fgf2*^−/−^, *n* = 3) suggesting no major abnormalities in the coronary anatomy. We then characterized cardiac vascular remodeling in *Fgf2*^−/−^ and wild‐type mice after IR injury by immunostaining for smooth muscle actin to identify smooth muscle containing medium and large vessels (Fig. 5A). In the peri‐infarct region, *Fgf2*^−/−^ hearts contained significantly fewer vessels per microscopic field compared to wild‐type hearts at 3 days and 7 days after IR injury (Fig. 5B, *P* < 0.05), and had significantly increased vessel diameter at 7 days after IR injury (Fig. 5C, *P* < 0.05). Capillary density and size were visualized in the peri‐infarct region by immunostaining for CD31 as a marker of endothelial cells (Fig. 7A). At 3 days and 7 days after IR injury, *Fgf2*^−/−^ hearts showed significantly reduced numbers of capillaries (normalized to nuclei number) compared to wild‐type hearts (Fig. 7B, *P* < 0.05), with no difference in average capillary size (Fig. 7C). Together, these data suggest a role for endogenous FGF2 in the post‐ischemic vascular remodeling response in the peri‐infarct region.

**Figure 8.**
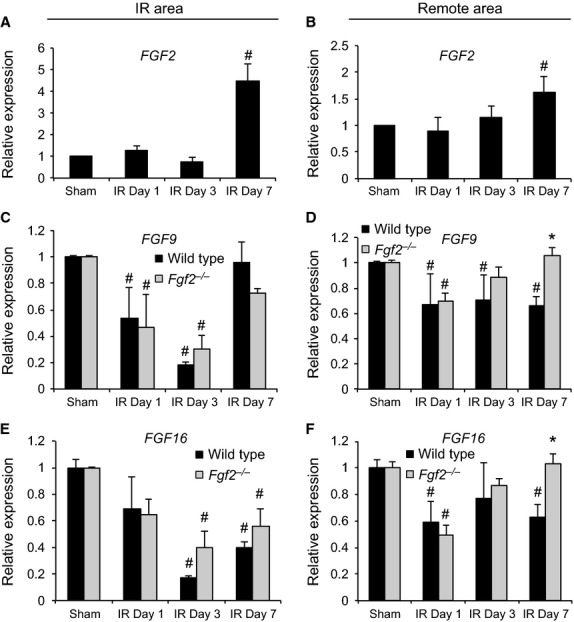
Regional FGF ligand expression by quantitative RT‐PCR in ischemia‐reperfusion injury. *Fgf2 *mRNA levels are significantly increased compared to sham controls in wild‐type mice 7 days after IR injury in both the IR area (A) and the remote area (B) of the left ventricle. *Fgf9 *mRNA levels are decreased after IR injury in a time‐dependent manner compared to sham controls in both wild‐type and *Fgf2*^−/−^ mice (C and D). Similarly, *Fgf16 *mRNA levels are decreased after IR injury (E and F). black bars – wild type, gray bars ‐ *Fgf2*^−/−^*, n* = 3–4, *P < 0.05 versus wild type, ^#^*P* < 0.05 versus sham.

**Figure 9.**
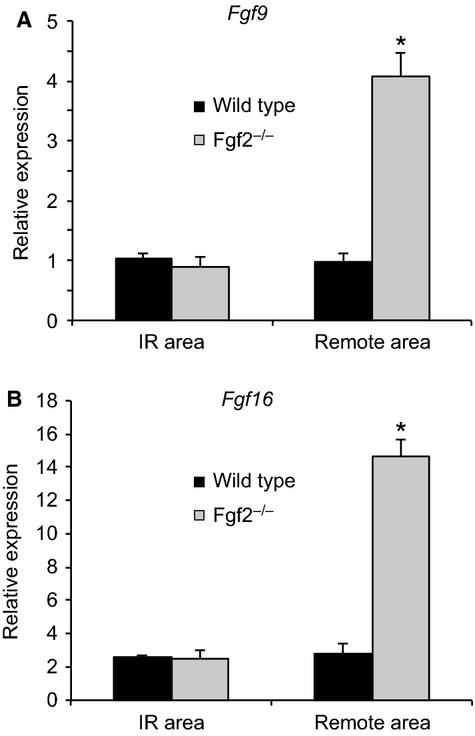
Regional FGF ligand expression by quantitative RT‐PCR at 7 days after ischemia‐reperfusion injury. *Fgf9* (A) and *Fgf16* (B) mRNA levels are significantly increased in the remote area but not the IR area of *Fgf2*^−/−^ hearts compared to wild‐type controls. *n* = 4, *P < 0.05 versus wild type.

In addition to *Fgf2*,* Fgf9*, and *Fgf16* are expressed in the adult heart (Fig. 8C–F) (Miyake et al. 1998; Wang et al. 2014). To determine whether *Fgf9* and *Fgf16* are coregulated with *Fgf2*, qRT‐PCR was performed. In the absence of injury (sham operated mice), mice lacking endogenous *Fgf2* and wild‐type control mice had nearly identical levels of *Fgf9* or *Fgf16* mRNA expression when normalized to *Gapdh* (*n* = 3–4, *P* < 0.05). Consistent with known increases in *Fgf2* expression following myocardial infarction (Zhao et al. 2011), *Fgf2* mRNA levels were significantly increased in wild‐type hearts at 7 days after IR injury in both the IR area and the remote area of the left ventricle (Fig. 8A and B, *P* < 0.05). In contrast, *Fgf9* mRNA levels were decreased at 1 day and 3 days after IR injury in both the IR and remote areas of wild‐type hearts (Fig. 8C and D, *P* < 0.05). *Fgf2*^−/−^ hearts showed a similar pattern in the IR area and decreased expression at day one in the remote area. Furthermore, in wild‐type hearts the remote area showed a decrease in *Fgf9* after IR injury that was sustained throughout the 7 days of reperfusion (Fig. 8D, #*P* < 0.05). Similar to *Fgf9*,* Fgf16* showed reduced expression at 1 day post‐IR in the remote area and at 3 days post‐IR in the IR area in both wild‐type and *Fgf2*^−/−^ hearts (Fig. 8E and F, #*P* < 0.05). Decreased expression of *Fgf16* was also observed in both wild‐type and *Fgf2*^−/−^ hearts in the remote area after IR injury (Fig. 8F, *P* < 0.05). At 7 days after IR injury, both *Fgf9* and *Fgf16* were significantly increased in *Fgf2*^−/−^ hearts compared to wild‐type hearts in the remote area (Fig. 8D and F, *P < 0.05). To control for observed small differences in *Hprt* expression in *Fgf2*^−/−^ hearts compared to wild‐type hearts at all time points, we also performed qRT‐PCR with normalization to *Gapdh*. Because *Gapdh* expression was not affected by lack of *Fgf2*, expression levels of *Fgf9* and *Fgf16* could be directly compared in wild‐type and *Fgf2*^−/−^ hearts. Seven days after IR injury, there was no difference in *Fgf9* and *Fgf16* expression, in the IR area, between wild‐type and *Fgf2*^−/−^ hearts (Fig. 9A and B). However, in the remote area, *Fgf2*^−/−^ hearts showed a fourfold overexpression of *Fgf9* and a fivefold overexpression of *Fgf16* compared to wild‐type controls (Fig. 9A and B, *P* < 0.05).

## Discussion

This study establishes the cardioprotective function of endogenous FGF2 in a clinically relevant model of closed‐chest regional IR injury. In the noninjured state, *Fgf2*^−/−^ mice have normal cardiac function as determined *ex vivo* by hemodynamic measurements (House et al. 2003) and in vivo by echocardiography (House et al. 2010b). Examination of acute postischemic cardiac function (1 day after IR injury) and longer term cardiac remodeling (7 days after IR injury) showed a significant decrease in cardiac function in *Fgf2*^−/−^ hearts compared to wild‐type controls at both time points. This is in agreement with studies showing reduced contractile functional recovery immediately after cardiac global low‐flow IR injury *ex vivo* (House et al. 2003).

Echocardiography was used to generate serial images that were subsequently analyzed for wall motion abnormalities. This echo method correlates well with histological measurement of infarct size in trichrome‐stained sections (Kanno et al. 2002). Comparing *Fgf2*^−/−^ and wild‐type hearts both acutely and chronically after IR injury showed an increased hypokinetic area in the absence of FGF2. In addition, we observed increased cell death in *Fgf2*^−/−^ mice acutely (1 day after IR injury) suggesting that FGF2 has an immediate cardioprotective effect on the myocyte. Previous studies of *Fgf2*^−/−^ hearts subjected to *ex vivo* IR injury did not show an effect on infarct size compared to wild‐type hearts (House et al. 2003; Liao et al. 2010). However, these investigations measured infarct size after only 2 h of reperfusion following global low‐flow ischemia in an isolated heart model, while here we measured infarct size 1 day and 7 days after IR injury in vivo. Another study evaluating *Fgf2*^−/−^ mice subjected to chronic coronary artery occlusion observed no difference in infarct size at their earliest time point (4 days after ischemia onset) but did observe a significantly increased infarct size in *Fgf2*^−/−^ mice beginning 1 week after the onset of ischemia, which was suggested to be primarily related to the necessity of FGF2 for infarct scar contraction during late remodeling (Virag et al. 2007). Differences between these findings and our observation of an acute cardioprotective requirement for FGF2 identifies a potential role for FGF2 in reducing myocyte cell death as a result of reperfusion injury in vivo.

FGF2 has been shown in multiple models to mediate cardiomyocyte hypertrophy (Schultz et al. 1999; Pellieux et al. 2001; House et al. 2010b). *Fgf2*^−/−^ hearts show no difference in heart weight to body weight ratios or myocyte cross‐sectional area in the absence of a stress stimulus (House et al. 2010b). By directly measuring myocyte cross‐sectional area, we identified a significant impairment in the cardiac hypertrophic response in the peri‐infarct area in *Fgf2*^−/−^ hearts, but no difference in the remote myocardium (left ventricular septum). This finding is in agreement with the observation that *Fgf2*^−/−^ mice show significantly reduced myocyte‐cross‐sectional area 4 weeks after permanent coronary occlusion compared to wild‐type controls (Virag et al. 2007) as well as other studies implicating FGF2 in the hypertrophic response to stimuli such as pressure overload, angiotensin, and isoproterenol (Schultz et al. 1999; Pellieux et al. 2001; House et al. 2010b).

While there is no difference between *Fgf2*^−/−^ and wild‐type mice with respect to vessel density in noninfarcted hearts (House et al. 2003), we found a significant reduction in the number of smooth muscle containing vessels in *Fgf2*^−/−^ mice after IR injury. The mean diameter of these vessels was also significantly larger in *Fgf2*^−/−^ hearts compared to wild‐type hearts. These data correlate with the findings of Virag et al. in the model of chronic cardiac ischemia (Virag et al. 2007). This difference in average vessel diameter is likely either due to increased loss of small vessels in the *Fgf2*^−/−^ hearts or increased growth of new low‐caliber vessels in the wild‐type hearts compared to *Fgf2*^−/−^ hearts after IR injury. Similarly, capillary density after IR was significantly reduced in *Fgf2*^−/−^ mice compared to wild‐type controls identifying a role for FGF2 in the normal process of vascular remodeling which occurs after cardiac IR injury.

Previous studies in isolated heart models have identified signaling pathways that may mediate the cardioprotective effects of FGF2. Increased expression of human FGF2 has been shown to reduce postischemic cardiac dysfunction and myocardial infarct size through activation of protein kinase C (PKC), mitogen‐activated protein kinase (MAPK), and nitric oxide signaling (House et al. 2005, 2007; Manning et al. 2012). Exogenous administration of recombinant FGF2 in an *ex vivo* model of cardiac ischemia‐reperfusion injury has also been shown to improve functional recovery and reduce apoptosis and was found to increase phosphorylation of AKT and p70 S6 kinase as well as translocation of PKC isoforms (Jiang et al. 2009). In vitro studies have shown that FGF2 increases resistance to calcium‐induced permeability transition pore opening in mitochondria, a known end‐effector in cardioprotective signaling (Srisakuldee et al. 2014). Future studies are necessary to determine the signaling mechanisms that mediate the cardioprotection provided by endogenous FGF2 in cardiac ischemia‐reperfusion injury in vivo.

Multiple FGF ligands are expressed in the adult heart and can be altered in various forms of cardiac pathology. We characterized the expression of *Fgf2*,* Fgf9*, and *Fgf16* at various time points following IR injury. As anticipated from previous studies, *Fgf2* mRNA levels were significantly increased in both the IR area and the remote area at 7 days after IR injury. This is also in agreement with a peak in FGF2 protein expression in both the infarct area and the border zone at 7 days after chronic LAD occlusion (Zhao et al. 2011). *Fgf9* and *Fgf16* encode two related FGFs that are important for cardiomyocyte growth during development (Lavine and Ornitz 2010; Ornitz and Itoh 2015). *Fgf16* is expressed at relatively high levels in the adult heart, while *Fgf9* is expressed at lower levels (Hall et al. 2004; Zhou et al. 2009). Interestingly, in wild‐type mice, both *Fgf9* and *Fgf16* were significantly decreased in the IR area during the first 3 days following IR injury. This decreased expression could reflect a physiological response to injury or result from differences in the cellular composition of the IR area following injury. Reduced expression of these *Fgfs* in the remote area of the heart, beginning 1 day after IR injury and continuing to day seven, supports a physiological regulation of *Fgf9* and *Fgf16* gene expression.

In *Fgf2*^−/−^ hearts, the relative levels of *Fgf9* and *Fgf16* expression returned to baseline levels 7 days after IR injury in the remote area of the heart. One hypothesis to explain this finding is that FGF2 negatively regulates *Fgf9* and *Fgf16* in the context of IR injury. Consistent with this hypothesis, *Fgf9* and *Fgf16* expression is significantly increased in the *Fgf2*^−/−^ mice compared to wild‐type controls at the time point when *Fgf2* is significantly increased in wild‐type hearts. Alternatively, the increase in *Fgf9* and *Fgf16* expression may represent a feedback mechanism to compensate for the absence of FGF2. Other studies have also observed interactions among these FGF ligands. In vitro, FGF16 abrogated FGF2‐induced cardiomyocyte proliferation, but did not alter proliferation in the absence of FGF2 (Lu et al. 2008). These data suggest that complex interactions may exist among FGF ligands in the heart during injury response and repair. Future studies will need to address the relative contribution of different FGF ligands and regulation among FGF ligands in the context of IR injury.

In conclusion, this study establishes the importance of endogenous FGF2 in protection from IR injury in vivo. In the absence of FGF2 after IR injury, there is reduced cardiac functional recovery, increased infarct size, impaired hypertrophic response, and impaired vascular remodeling. Further, this investigation has utilized a model of cardiac IR injury that more closely recapitulates the clinical scenario of acute myocardial infarction.

## Acknowledgments

The authors thank Traian Stefan Lupu for excellent technical assistance.

## Conflict of Interest

None declared.
